# Repositive RT-PCR test in discharged COVID-19 patients during medical isolation observation

**DOI:** 10.7150/ijms.58766

**Published:** 2021-04-26

**Authors:** Kun Huang, Wen Liu, Jinxia Zhou, Yao Wang, Yuxiang Zhang, Xinle Tang, Jing Liang, Fang-Fang Bi

**Affiliations:** 1 Department of Neurology, Xiangya Hospital, Central South University, Changsha, Hunan province, China.; 2 Department of laboratory medicine, the Sixth Affiliated Hospital of Xinjiang Medical University, Urumqi, Xinjiang Uygur Autonomous Region, China.

**Keywords:** Coronavirus disease 2019 (COVID-19), discharged patient, real-time PCR, repositive, antibody

## Abstract

**Objectives:** The epidemiological and clinical characteristics of patients with coronavirus disease 2019 (COVID-19) have been researched. However, the prevalence of repositivity by real-time PCR for severe acute respiratory syndrome coronavirus 2 (SARS-CoV-2) remains unclear.

**Methods:** A retrospective study was conducted involving 599 discharged patients with COVID-19 in a single medical centre. The clinical features of patients during their hospitalization and 14-day post-discharge quarantine were collected.

**Results:** A total of 122 patients (20.4%) out of 599 patients retested positive after discharge. Specifically, 94 (15.7%) retested positive within 24 h of discharge, and another 28 patients (4.7%) were repositive on day 7 after discharge, although none showed any clinical symptomatic recurrence. Both repositives and non‑repositives have similar patterns of IgG and IgM. Notably, the length of hospitalization of non-repositive patients was longer than that of 24-h repositive patients and 7-day repositive patients. In addition, the length of hospitalization of 24-h repositive patients was shorter than that of 7-day repositive patients, indicating that the length of hospitalization was also a determinant of viral shedding.

**Conclusion:** Our study provides further information for improving the management of recovered and discharged patients, and further studies should be performed to elucidate the infectiveness of individuals with prolonged or RNA repositivity.

## Introduction

Coronavirus disease 2019 (COVID-19) is an emerging acute respiratory infectious disease caused by severe acute respiratory syndrome coronavirus 2 (SARS-CoV-2) and has spread worldwide 1. COVID-19 is a highly infectious disease that has been announced by the World Health Organization (WHO) as having reached pandemic status 2. The main clinical manifestations are fever, cough and fatigue; the degree of infection ranges from asymptomatic, mild, and moderate to death 3, 4. As of Mar 22, 2021, more than 716,000 deaths and 123,287,000 confirmed positive cases have been reported worldwide.

With global efforts, the number of cured patients has increased. The COVID-19 pandemic is becoming complex and difficult to control in terms of both morbidity and mortality rates. The pandemic is epidemic in most countries worldwide, even developed countries with modern and advanced medical systems. Some developing countries, such as Brazil and India, are facing an increasing number of cases without a decreasing trend since the first case. Moreover, increasing problems have occurred in the follow-up and re-examination of recovered patients after discharge.

Currently, most researchers are focusing on the epidemiological characteristics of COVID-19 patients, as well as the clinical manifestations and efficacy outcomes. However, limited studies have been conducted on patients who have recovered and been discharged, which has significantly affected our complete understanding of the disease. The phenomenon of repositive real-time reverse transcription polymerase chain reaction (RT-PCR) results of SARS-CoV-2 in recovered patients has occurred recently 5, 6. From July 15, 2020, Urumqi had a COVID-19 pandemic after 149 days without new cases. In this research, we retrospectively analysed the clinical data and laboratory characteristics of 599 discharged patients with COVID-19 from Xinjiang Uygur Autonomous Region, which is in the northwest part of China, and observed that a certain number of discharged patients showed repositive RT-PCR results. Our study will provide a reference for the management and follow-up of the COVID-19 pandemic.

## Methods

### Patients

This retrospective study was conducted at the Sixth Affiliated Hospital of Xinjiang Medical University, China. Patients infected with COVID-19 were divided into severe and non-severe (mild and moderate) groups according to the guidelines for “Diagnosis and Treatment of Pneumonia Caused by Novel Coronavirus (Trial Version 7)” 7. Briefly, the severe patients were individuals who met any of the following criteria: respiratory failure and requiring mechanical ventilation; shock; or with other organ failure that requires ICU care. Non-severe patients were the other COVID-19 patients. All non-severe patients hospitalized in the Sixth Affiliated Hospital of Xinjiang Medical University were enrolled from July 15, 2020, when Urumqi started to have a COVID-19 pandemic, to Oct 22, 2020, with the approval of the Ethics Committee of the Sixth Affiliated Hospital of Xinjiang Medical University (No. LFYLLSC20210107-01). After written informed consent was obtained from each patient, a total of 599 identified Chinese COVID-19 patients who met the discharge criteria were recruited. The discharge criteria were as follows: (1) body temperature below 37 °C, lasting for at least three consecutive days; (2) resolved respiratory symptoms; (3) obvious amelioration on computed tomography (CT) images; and (4) two consecutive negative real-time PCR test results of nasopharyngeal swabs > 24 h apart 7. According to the local policy, all the discharged patients were asked to stay in the medical isolation centre for an additional 14 days (a single room for every patient) to avoid cross-infection.

### Real-time PCR examination

The results of this study were analysed and reported in accordance with the STROBE guidelines 8. The real-time PCR test was performed as previously described with modification 9, 10, and was based on the criteria provided by the World Health Organization (WHO) 11. Nasopharyngeal swabs were taken on the 1st, 7th and 14th days of medical isolation observation. Repositive patients were tested every day until turn to negative for consecutive two days during medical isolation.

The nucleic acid kit, Daan novel coronavirus nucleic acid detection kit (real-time PCR kit, Da An Gene Co., Ltd. of Sun Yat-sen University, China) was recommended by the Chinese Center for Disease Control and Prevention (CDC), and extraction of nucleic acids from clinical samples (including negative controls) was performed as described by the manufacturer. To ensure that the real-time PCR results were accurate, the same sample was also checked using another nucleic acid kit, Liferiver novel coronavirus nucleic acid detection kit, following the instructions of the manufacturer (Shanghai ZJ Bio-Tech Co., Ltd., China). The limit of detection was defined as the lowest concentration level that can be detected with a specified kit. The limit of detection according to the manufacturer of each kit was 500 copies/mL for the Daan kit and 1000 copies/mL for the Liferiver kit. According to a previous study, these two nucleic acid detection kits have similar quality. The sensitivity was 100% for the Daan kit and 90% for the Liferiver kit. The specificity was 100% for both of these kits 12.

Two target genes of SARS-CoV-2, including open reading frame 1ab (ORF1ab) and nucleocapsid protein (N), were simultaneously amplified and tested using the real-time PCR assay. Any of the positive results for ORF1ab or N were determined to be positive. Target 1 (ORF1ab): forward primer: 5'-CCCTGTGGGTTTTACACTTAA-3'; reverse primer: 5'-ACGATTGTGCATCAGCTGA-3'; probe: 5′-VIC-CCGTCTGCGGTATGTGGAAAGGTTATGG-BHQ1-3′. Target 2 (N): forward primer: 5'-GGGGAACTTCTCCTGCTAGAAT-3'; reverse primer: 5'-CAGACATTTTGCTCTCAAGCTG-3'; probe: 5′-FAM-TTGCTGCTGCTTGACAGATT-TAMRA-3′. A cycle threshold value (Ct-value) less than 37 was defined as positive, and a Ct-value no less than 40 was defined as negative. Weakly positive was defined as 37 ≤ Ct < 40, and this value was further confirmed by retesting. A positive result was reported if a Ct-value ≤ 40 was reported from the retest on the next day. If the results of the Daan kit and the Liferiver kit were discordant, a positive result for either the Daan kit or the Liferiver kit was reported as positive, while a negative result for both the Daan kit and the Liferiver kit was deemed negative.

### Antibody detection

Antibody detection was carried out on the first day after discharge. IgG and IgM were tested using the SARS-CoV-2 testing kit (AutoLumoA2000Plus, Autobio Diagnostics Co. Ltd., China) based on the chemiluminescence method, targeting the SARS-CoV-2 spike antigen. All tests were performed according to the manufacturer's instructions. According to the manufacturer's instructions, the IgG sensitivity and specificity were 93.3% and 98.8%, respectively, and the IgM sensitivity and specificity were 50.0% and 100%, respectively.

### Data collection

Data on demographic and clinical characteristics and course of disease were extracted. Symptoms of the observed subjects in and out of the medical isolation centre and comprehensive intervention were also recorded. If the relevant information was missing, we directly contacted the patient's family.

### Statistical analysis

Numerical variables were summarized as median with interquartile range (IQR). The categorical variables are described as counts and percentages. The Pearson chi-square (χ^2^) test was used to compare the frequency distribution of categorical variables. The Mann-Whitney U test or Kruskal-Wallis test was used to compare the difference among different groups as previously used 13. The statistical analysis was performed using the Statistical Package for Social Sciences (SPSS Inc., Chicago, IL, USA) Version 20. A *p*-value <0.05 was considered statistically significant.

## Results

### Demographic and clinical characteristics

A total of 599 identified COVID-19 patients recovered and were discharged. Four hundred forty-nine patients (75.0%) were Uyghur individuals, and 106 patients (17.7%) were Han Chinese individuals. The median age of the patients was 34.0 (IQR 22.0-48.0) years. Two hundred and sixty-eight (44.7%) patients were males, and 331 (55.3%) were females. Since the National Health Commission of China recommended the Lian-Hua Qing-Wen Granule, a traditional Chinese medicine, as a therapeutic drug in the guidelines for the treatment of COVID-19 7, all patients were prescribed the Lian-Hua Qing-Wen Granule after diagnosis. Nonsteroidal anti-inflammatory drugs, such as ibuprofen and acetaminophen, were only used in patients whose body temperatures were over 38.0 °C. No other medications were given. The median length of hospitalization of the 599 patients was 34.0 (IQR 15.0-34.0) days. The demographic characteristics are shown in **Table 1**.

### Repositive patients

COVID-19 patients from the Sixth Affiliated Hospital of Xinjiang Medical University who met all of the hospital discharge criteria were recommended to stay in a medical centre with strict medical isolation for an additional 14 days. During the 14-day isolation, all the discharged patients were retested for SARS-CoV-2 with nasopharyngeal swabs by real-time PCR at day 1, day 7 and day 14. In addition, if patients were tested repositive during medical isolation, these patients were retested every day until they tested negative twice consecutively. During medical isolation after discharge, positive real-time PCR results were reported as repositive.

Ninety-four patients (15.7%) out of 599 patients retested positive within 24 h of discharge. Thirty-nine (41.5%) were males, and 55 (58.5%) were females. The median age was 33.0 (IQR 24.0-47.0) years. The median length of hospitalization before discharge was 16.0 (IQR 14.0-19.0) days. Among all the positive retest patients, 70 patients were asymptomatic, and the remaining positive retest patients showed slight symptoms such as fatigue, sneezing and high normal temperature (lower than 37.4 °C). All the symptomatic repositive patients did not worsen after treatment with Lian-Hua Qing-Wen granules. The retest patients were retested every day after repositivity. The median length of time before the positive retest patients became real-time PCR negative was 4.0 (IQR 3.0-5.0) days **(Table 1)**.

**Figure 1:**
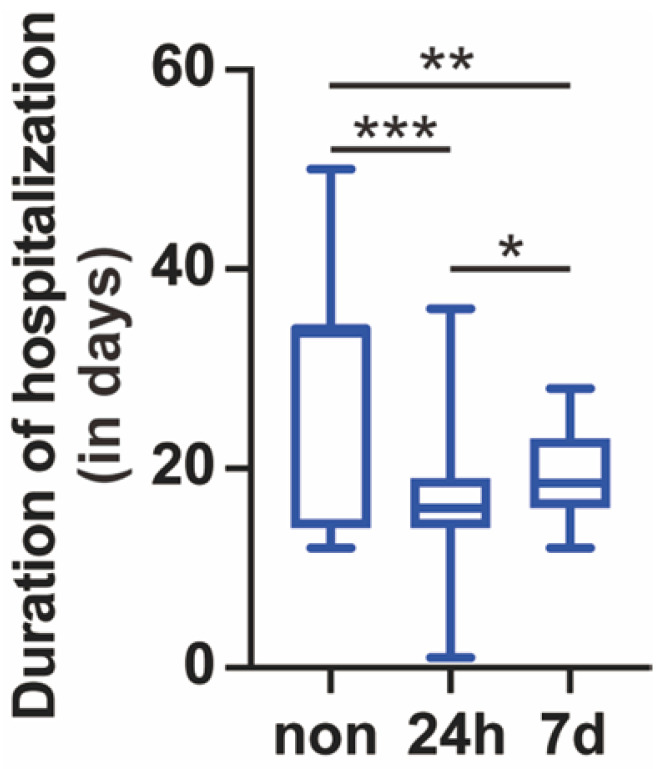
** Length of hospitalization.** The length of hospitalization of negative retest, 24-h positive retest, and 7-day positive retest patients is shown. Nonrepositive patients had a longer hospitalization than 24-h repositive and 7-day repositive patients (*p* < 0.001 and 0.01, respectively). Twenty-four-hour repositive patients had a shorter hospitalization than 7-day repositive patients (*p* < 0.05). The median, quantile and extreme values were indicated. *p < 0.05, **p < 0.01, and ***p < 0.001 by the Kruskal-Wallis test followed by Dunn's multiple comparison test.

**Figure 2:**
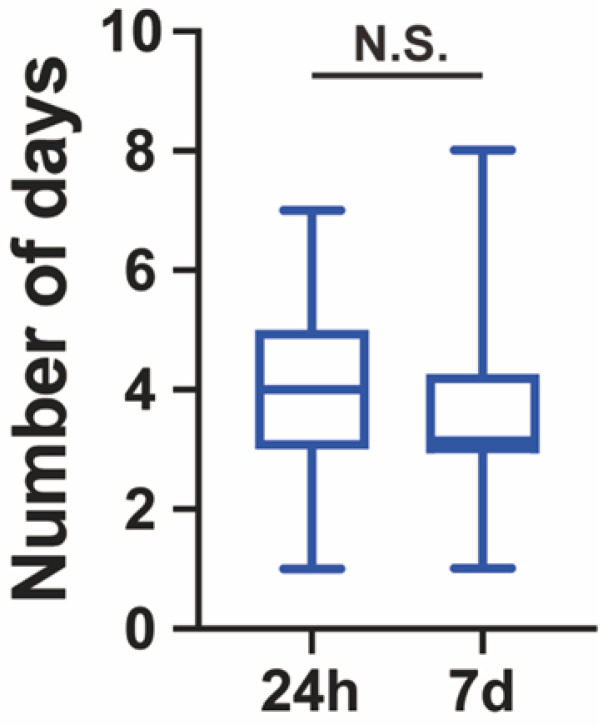
** Number of days required for the repositive patients to become negative.** All the discharged patients were retested for SARS-CoV-2 at day 1, day 7 and day 14. If patients were tested repositive, these patients were retested every day until they tested negative twice consecutively. No significant differences between the time required for the 24-h repositive and 7-day repositive patients to become negative (*p* = 0.051). The median, quantile and extreme values are indicated. N.S., not significant by the Mann-Whitney U test.

Another 28 patients (4.7%) were repositive on day 7 after discharge. Fourteen (50.0%) were males, and 14 (50.0%) were females. The median age was 23.0 (IQR 17.5-43.5) years. The median length of hospitalization before discharge was 3.0 (IQR 3.0-4.0) days. The length of hospitalization for 24-h repositive was shorter than that for 7-day repositive **(Figure 1)**. Among the positive retest patients, all were asymptomatic. The median length of time before the positive retest patients became real-time PCR negative was 4.0 (IQR 3.0-5.0) days **(Table 1)**. The length of time for repositive patients to become negative was not significantly different between the 24-h repositive patients and 7-day repositive patients **(Figure 2)**.

### Non-repositive patients

A total of 477 patients were negative upon retest during further isolation. Two hundred and fifteen (45.1%) were males, and 262 (54.9%) were females. The median age was 35.0 (IQR 22.0-49.0) years. The median length of hospitalization before discharge was 34.0 (IQR 14.0-34.0) days **(Table 1)**. The length of hospitalization of non-repositive patients was longer than that of 24-h repositive patients and 7-day repositive patients **(Figure 1)**. Furthermore, the repositivity was correlated with the length of hospitalization before discharge (Spearman's correlation coefficient, *r* = -0.28, *p* < 0.001).

### Re-positives and non-repositives have similar patterns of IgG and IgM

All the 599 discharged patients were tested for IgG and IgM on the first day after admission into the isolation centre. There were no significant differences in IgG and IgM patterns between the 122 repositive recovered COVID-19 patients and the 477 non-repositive patients **(Table 2).**

## Discussion

In the current research, we found that 20.4% of discharged COVID-19 patients still tested positive after discharge, which is in line with the previously reported incidence of positivity in patients with recurrent SARS-CoV-2 RNA 14. Although several single centres have already reported positive results in retested recovered COVID-19 patients, the current study has some unique characteristics 15. First, to our knowledge, this is the largest cohort for the repositive patients. Second, unlike other studies, our discharged patients were strictly medically isolated in a medical centre, which excluded the possibility of reinfection 16. Third, to minimize the mistake of real-time PCR detection techniques, all patients received double-checked tests with two different approved detection kits (one from Da An Gene Co., Ltd. of Sun Yat-sen University and another from Shanghai ZJ Bio-Tech Co., Ltd.). Our research provides a more accurate rate of repositivity in discharged COVID-19 patients.

None of the 122 positive retest patients showed any recurrence of typical COVID-19-related clinical symptoms, such as fever, cough or respiratory tract disease. These data indicate that the repositive phenomenon is likely due to the presence of virus residues in the body during recovery. Besides, debris of the virus may also be a possible reason for repositivity. The repositivity of SARS-CoV-2 could be a sign of active viral replication, and the repositive patients may still be infectious, although the infectiousness of repositivity requires further evaluation.

The observed length of hospitalization in this study was longer than that in some previous studies 17-19. The following reasons may explain this observation. First, the Chinese government asked all people from Urumqi to undergo a PCR screening test after Urumqi experienced the COVID-19 pandemic. Because of the different policies of different governments, almost 85% of the patients enrolled in our current study were diagnosed by PCR screening after the first patient was found in Urumqi, when the enrolled patients were still at a very early stage of disease without obvious symptoms. COVID-19 patients have similar clinical manifestations as those with other viral infections, such as fever, cough, and fatigue, leading to a delay in visiting a doctor during early illness 20. Indeed, the median duration of time from illness onset to admission was 11 (IQR 8-14) days 21, leading to a relatively longer length of hospitalization in our study.

Second, a systematic review of COVID-19 hospitalizations that analysed 41 studies from China and four studies from the rest of the world 22 reported that the length of hospitalization was much longer for patients in China than for patients in the rest of the world, which is probably attributable to the fact that other countries set less strict criteria for hospitalization and discharge. Third, we could not totally exclude the possibility that some of our patients may be immunocompromised, which may prolong the length of hospitalization. Although all the patients from our study did not receive a transplant and were not using immunosuppressors, we did not examine whether they had malignant cancer or HIV. Fourth, comorbidity also affects the length of hospitalization. Patients with comorbidities have a longer length of hospitalization 23. In our current study, 52 patients (8.7%) had diabetes mellitus or hypertension, which was relatively lower than previous report, partly due to the omission of other comorbidities. The neglected comorbidities may partly explain the longer length of hospitalization. Last, we diagnosed RNA positivity according to the viral RNA cycle threshold value, which did not distinguish dead viruses from active viruses.The Ct-value ≤ 40 were reported as positive in our study, specifically, the 37 ≤ Ct < 40 may not be able to detect the infectious virus and may lead to false-positive results, partially explaining the long duration of positivity.

The reasons for repositivity after discharge remain unclear. There are several possibilities. First, viruses severely damage the immune function of patients, especially when accompanied by comorbidities in patients, so the virus is not completely cleared 24. Second, since no antiviral drug was used, and the effect of the traditional Chinese medicine, Lian-Hua Qing-Wen Granule, might be limited, so virus replication was only partly inhibited 21. Third, the duration of SARS-CoV-2 RNA detection has not been well characterized, and its duration may be longer than that currently known 25.

Previous studies have reported several factors that prolong viral shedding 26. One systematic review and meta-analysis concluded that corticosteroid treatment was associated with delayed viral clearing 27. Another study found that clearance of SARS-CoV-2 took a longer time in paediatric patients with gastrointestinal disease than in those with only respiratory disease 28. In the current study, we found that the length of hospitalization was also a determinant of viral shedding, which should be considered in discharge timing.

Due to technique limitations, we could only identify anti-SARS-CoV-2 antibodies as positive or negative. Previous studies detected that the median seroconversion time of IgM and IgG against SARS-CoV-2 varies differently, ranging from 5 to 13 days, 11 to 14 days, respectively, after symptom onset 29. In this study, we found that both repositive and nonrepositive patients had similar patterns of IgG and IgM, which is in accordance with previous reports. Yuan *et al*. analysed the total antibody, IgM, IgG, and IgA, but no significant differences between repositive recovered COVID-19 patients and nonrepositive patients were found 5. Furthermore, they did not find an association between viral load and antibody titre. Our research, together with previous research, suggests that the repositive patients shed viral RNA segments, and repositive patients were not associated with antibody level fluctuations.

There are several limitations in the present study. First, all the enrolled patients in our study had mild or moderate symptoms of COVID-19 during hospitalization and none showed severe symptoms during hospitalization. Second, further follow-up studies, such as those that involve more than 14 days of isolation, need to be conducted among these patients to elucidate the possible mechanisms of the repositive phenomenon. Third, all patients in this study were from monocentre. Multicentre research on a larger cohort is needed in future studies.

## Conclusions

In summary, our research found that a proportion of discharged patients still can have a positive result by real-time PCR test of SARS-CoV-2. Further studies are still important to elucidate the infectiveness of individuals who are RNA repositive.

## Figures and Tables

**Table 1:** Clinical features of the patients enrolled in the study

	Number	Age (years) (median, IQR)	Male:Female	Length of hospitalization (days) (median, IQR)	Days of turn to negative^a^ (median, IQR)
Total patients	599	34.0 (22.0-48.0)	268:331	34.0 (15.0-34.0)	-
24-h repositive	94	33.0 (24.0-47.0)	39:55	16.0 (14.0-19.0)	4.0 (3.0-5.0)
7-day repositive	28	23.0 (17.5-43.5)	14:14	18.5 (16.0-23.0)	3.0 (3.0-4.0)
Repositive	122	32.0 (21.0-47.0)	53:69	17.0 (15.0-21.0)	4.0 (3.0-5.0)
Non-repositive	477	35.0 (22.0-49.0)	215:262	34.0 (14.0-34.0)	-

^a^Days of turn to negative: Days of repositive patients from real-time PCR repositive to become negative during medical isolation.

**Table 2:** Antibody detection of IgG in positive retest SARS-CoV-2 viral RNA patients and negative retest patients

	IgG Positive	IgG Negative	Percent positive (%)	IgM Positive	IgM Negative	Percent positive (%)
Repositive	114	8	93.4	85	37	69.7
Non-repositive	427	50	89.5	335	142	70.2
*p*-value			0.191			0.906

## References

[1] Huang C, Wang Y, Li X, Ren L, Zhao J, Hu Y (2020). Clinical features of patients infected with 2019 novel coronavirus in Wuhan, China. Lancet.

[2] WHO. 2020

[3] Chen N, Zhou M, Dong X, Qu J, Gong F, Han Y (2020). Epidemiological and clinical characteristics of 99 cases of 2019 novel coronavirus pneumonia in Wuhan, China: a descriptive study. Lancet.

[4] Xu XW, Wu XX, Jiang XG, Xu KJ, Ying LJ, Ma CL (2020). Clinical findings in a group of patients infected with the 2019 novel coronavirus (SARS-Cov-2) outside of Wuhan, China: retrospective case series. BMJ.

[5] Yuan B, Liu HQ, Yang ZR, Chen YX, Liu ZY, Zhang K (2020). Recurrence of positive SARS-CoV-2 viral RNA in recovered COVID-19 patients during medical isolation observation. Sci Rep.

[6] Cao H, Ruan L, Liu J, Liao W (2020). The clinical characteristic of eight patients of COVID-19 with positive RT-PCR test after discharge. J Med Virol.

[7] China National Health Commission Diagnosis and treatment of 2019-nCoV pneumonia in China. (Version 7).

[8] von Elm E, Altman DG, Egger M, Pocock SJ, Gotzsche PC, Vandenbroucke JP (2008). The Strengthening the Reporting of Observational Studies in Epidemiology (STROBE) statement: guidelines for reporting observational studies. J Clin Epidemiol.

[9] Huang K, Li J, Ito M, Takeda JI, Ohkawara B, Ogi T (2020). Gene Expression Profile at the Motor Endplate of the Neuromuscular Junction of Fast-Twitch Muscle. Front Mol Neurosci.

[10] Huang K, Masuda A, Chen G, Bushra S, Kamon M, Araki T (2020). Inhibition of cyclooxygenase-1 by nonsteroidal anti-inflammatory drugs demethylates MeR2 enhancer and promotes Mbnl1 transcription in myogenic cells. Sci Rep.

[11] World Health Organization (2019). (2020). Coronavirus disease.

[12] Wang M, Chen D, Wu W, Tang H, Kan L, Zong Z (2021). Analytical performance evaluation of five RT-PCR kits for severe acute respiratory syndrome coronavirus 2. J Clin Lab Anal.

[13] Huang K, Luo YB, Bi FF, Yang H (2020). Pharmacological strategy for congenital myasthenic syndrome with CHRNE mutations: a meta-analysis of case reports. Curr Neuropharmacol.

[14] Azam M, Sulistiana R, Ratnawati M, Fibriana AI, Bahrudin U, Widyaningrum D (2020). Recurrent SARS-CoV-2 RNA positivity after COVID-19: a systematic review and meta-analysis. Sci Rep.

[15] Lan L, Xu D, Ye G, Xia C, Wang S, Li Y (2020). Positive RT-PCR Test Results in Patients Recovered From COVID-19. JAMA.

[16] Zhou J, Zhang J, Zhou J, Yi H, Lin Z, Liu Y (2020). Clinical characteristics of re-positive COVID-19 patients in Huangshi, China: A retrospective cohort study. PLoS One.

[17] Bhatraju PK, Ghassemieh BJ, Nichols M, Kim R, Jerome KR, Nalla AK (2020). Covid-19 in Critically Ill Patients in the Seattle Region - Case Series. N Engl J Med.

[18] Shu HM, He S, Sun Y, Lin CQ, Lu YF, Liu J (2021). Factors Influencing Viral Clearance in Mild COVID-19 and Clinical Characteristics of Asymptomatic Patients. Biomed Res Int.

[19] Qiu H, Wu J, Hong L, Luo Y, Song Q, Chen D (2020). Clinical and epidemiological features of 36 children with coronavirus disease 2019 (COVID-19) in Zhejiang, China: an observational cohort study. Lancet Infect Dis.

[20] Rubin EJ, Baden LR, Morrissey S (2020). Audio Interview: New Research on Possible Treatments for Covid-19. N Engl J Med.

[21] Zhou F, Yu T, Du R, Fan G, Liu Y, Liu Z (2020). Clinical course and risk factors for mortality of adult inpatients with COVID-19 in Wuhan, China: a retrospective cohort study. Lancet.

[22] Rees EM, Nightingale ES, Jafari Y, Waterlow NR, Clifford S, CA BP (2020). COVID-19 length of hospital stay: a systematic review and data synthesis. BMC Med.

[23] Dong G, Du Z, Zhu J, Guo Y, Gao W, Guo W (2021). The clinical characteristics and prognosis of COVID-19 patients with comorbidities: a retrospective analysis of the infection peak in Wuhan. Ann Transl Med.

[24] Ling Y, Xu SB, Lin YX, Tian D, Zhu ZQ, Dai FH (2020). Persistence and clearance of viral RNA in 2019 novel coronavirus disease rehabilitation patients. Chin Med J (Engl).

[25] Xu D, Zhang Z, Jin L, Chu F, Mao Y, Wang H (2005). Persistent shedding of viable SARS-CoV in urine and stool of SARS patients during the convalescent phase. Eur J Clin Microbiol Infect Dis.

[26] Yan D, Liu XY, Zhu YN, Huang L, Dan BT, Zhang GJ (2020). Factors associated with prolonged viral shedding and impact of lopinavir/ritonavir treatment in hospitalised non-critically ill patients with SARS-CoV-2 infection. Eur Respir J.

[27] Li H, Chen C, Hu F, Wang J, Zhao Q, Gale RP (2020). Impact of corticosteroid therapy on outcomes of persons with SARS-CoV-2, SARS-CoV, or MERS-CoV infection: a systematic review and meta-analysis. Leukemia.

[28] Xing YH, Ni W, Wu Q, Li WJ, Li GJ, Wang WD (2020). Prolonged viral shedding in feces of pediatric patients with coronavirus disease 2019. J Microbiol Immunol Infect.

[29] Fu Y, Pan Y, Li Z, Li Y (2020). The Utility of Specific Antibodies Against SARS-CoV-2 in Laboratory Diagnosis. Front Microbiol.

